# Down-regulation of miR-125b by HPV16 E6 might promote cervical cancer progression through TAZ/TEAD

**DOI:** 10.3389/fonc.2025.1444874

**Published:** 2025-04-15

**Authors:** Yongli Hou, Lili Zhang, Wenhao Wang, Keyan Cheng, Hui Wang, Yanfei Ji, Xiaoqiang Su, Min Hao

**Affiliations:** Department of Gynecology and Obstetrics, The Second Hospital of Shanxi Medical University, Taiyuan, Shanxi, China

**Keywords:** cervical cancer, Hippo pathway, HPV16 E6, miR-125b, TAZ

## Abstract

**Background:**

Abnormal gene expression due to the dysregulation of microRNAs (miRNAs) often occurred in the initiation or progression of cancers. In this attempt, we investigate whether miR-125b regulates biological behaviors of cervical cancer caused by HPV16 through TAZ.

**Methods:**

Through the application of bioinformatics analysis techniques, differentially expressed miRNAs relevant to cervical cancer were identified. Cervical tissue specimens were collected from 15 patients with HPV16-positive cervical squamous cell carcinoma (stages IA-IIA), 15 patients with high-grade squamous intraepithelial lesion (HSIL), and 10 patients experiencing chronic cervical inflammation at the Second Hospital of Shanxi Medical University between May 2022 and May 2023. The quantitative assessment of miR-125b expression was conducted via real-time quantitative reverse transcription PCR(RT-qPCR). The potential regulatory relationship between miR-125b and TAZ was assessed using a dual-luciferase reporter assay. Within cervical squamous cell carcinoma SiHa cells, models were established using miR-125b mimic and inhibitor constructs to scrutinize cellular physiological processes and assess the expression profiles of miR-125b, TAZ, TEAD, and E6. Additionally, an HPV16 E6 overexpression cellular model was generated, and the expression patterns of miR-125b and its downstream targets were analyzed by RT-qPCR.

**Results:**

Tissue validation corroborated these findings, demonstrating a significant reduction in miR-125b expression levels in HSIL and cervical squamous cell carcinoma compared to normal cervical tissue (*P* < 0.05). Functional assays using a miR-125b mimic revealed inhibited proliferation, migration, and invasion of SiHa cells along with enhanced apoptosis, concomitant with decreased expression of HPV16 E6, TAZ, and TEAD mRNA. Conversely, these effects were reversed upon introduction of a miR-125b inhibitor. Notably, overexpression of HPV16 E6 was associated with suppressed miR-125b expression (*P* < 0.05) and enhanced TAZ and TEAD expression (*P* < 0.05), as corroborated by western blot analysis.

**Conclusion:**

HPV E6 promotes the malignant progression of cervical cancer cells by downregulating miR-125b, which targets TAZ, thus regulating the Hippo pathway. Consequently, miR-125b emerges as a promising therapeutic target for HPV-induced cervical cancer.

## 1 Introduction

Cervical cancer continues to pose a significant global public health challenge despite international efforts targeting its elimination. Despite widespread clinical implementation of screening programs and prophylactic vaccines, the incidence and mortality rates, particularly in developing nations, have not decreased notably (1). In China, the age-standardized incidence rate (ASIR) and age-standardized mortality rate (ASMR) for cervical cancer have exhibited a consistent upward trend, escalating from 3.79 per 100,000 and 1.44 per 100,000 in 2000 to 11.37 per 100,000 and 3.39 per 100,000 in 2016, respectively, underscoring a persistent and formidable disease burden (2).

It is widely acknowledged that persistent infection with high-risk human papillomavirus (HR-HPV), is the primary risk factor for cervical cancer. Among them, HPV16 is the most common, which accounts for approximately 50% of all cases (3). The entire HPV genome randomly integrates into the host genome once the HPV episomal genome is linearized, breaking particularly at the E1 and E2 genes, allowing for continued expression of the viral oncoproteins E6 and E7. They interfere with the normal cell growth and apoptosis regulation by interacting with the proteins of host cells, thus promoting cell carcinogenesis. However, despite over 80% of women experiencing HPV infection at some point in their lives, the progression from normal cervical epithelium to cervical intraepithelial neoplasia (CIN) and ultimately to cervical cancer is a prolonged process, with less than 1% of infections leading to cancer (4). Hence, the precise molecular mechanisms underlying HPV-induced cervical cancer remain elusive, with the E6 and E7 oncoprotein potentially contributing to disease initiation and progression through diverse pathways.

Recent investigations indicate that HPV16 E6 in dimeric form can regulate YAP/TAZ expression, activating the Hippo pathway, which in turn fosters tumor cell proliferation, impedes apoptosis, and inhibits the innate immune response of the host cell, thereby facilitating persistent HPV infection (5, 6). Consequently, targeting the E6 oncoprotein-mediated Hippo pathway represents a promising strategy to prevent the progression of HPV infections to cervical cancer.

The Hippo pathway, comprising of a series of protein kinases and transcription factors, is highly conserved in mammals. Dysregulation or hyperactivation of this pathway can result in aberrant cell growth and disruptions in tissue and organ homeostasis, ultimately contributing to tumorigenesis (7). The Hippo pathway is a conserved signaling cascade that regulates tissue homeostasis, cell proliferation, and apoptosis. Its core components—MST1/2 and LATS1/2 kinases—phosphorylate and inactivate the oncogenic co-activators YAP and TAZ, promoting their cytoplasmic retention and degradation. When Hippo signaling is disrupted, YAP/TAZ translocate to the nucleus, bind TEAD transcription factors, and drive expression of genes supporting cell growth and survival. Dysregulation of this pathway contributes to various cancers, including cervical carcinogenesis. TAZ, a pivotal transcriptional co-activator and key effector of the Hippo signaling pathway, is involved in regulating cellular proliferation, organ size regulation, and tumor progression. Abudoukerimu et al. confirmed through tissue and cellular investigations that with increasing severity of cervical lesions, TAZ expression levels escalate, indicating its oncogenic role in cervical cancer, although the precise mechanism remains unclear (8). Recent findings have elucidated the significant involvement of certain microRNAs (miRNAs) in modulating the onset and progression of tumors by targeting the Hippo pathway. For instance, in breast cancer, miR-326 targets TAZ, and its overexpression significantly reduces TAZ mRNA and protein levels, thereby inhibiting cell proliferation and migration while promoting apoptosis (9). Similarly, Tan et al. discovered that miR-129-5p directly suppresses TAZ mRNA and protein expression in ovarian cancer, resulting in the deactivation of TEAD transcription and impeding ovarian cancer progression (10). However, reports on the regulatory role of miRNAs in modulating TAZ expression in cervical cancer remain scarce.

miRNAs are one of the shortest endogenous noncoding RNAs, consisting of 20–25 nucleotides exert regulatory control over gene expression at the post-transcriptional level (11). Studies underscore their involvement in various cellular processes and highlight their use as biomarkers for cancer diagnosis and treatment. In cervical cancer, miRNAs exhibit dual roles as oncogenes or tumor suppressor genes, influencing HPV infection and various epigenetic modifications, including DNA methylation processes (12). Jianing Zhang found that HPV16 E6 promoting cervical cancer progression through down-regulation of miR-320a to increase TOP2A expression (13). Jing Hu et al. also found that The HPV16 E6, E7/miR-23b-3p/ICAT signaling axis promotes proliferation, migration, invasion and EMT of cervical cancer cells (14). And Bao Zang etal reveals that HPV-16 E6 promotes cell growth of esophageal cancer via downregulation of miR-125b and activation of Wnt/β-catenin signaling pathway (15).Therefore, investigating miRNAs that modulate TAZ, influenced by E6 in cervical cancer, holds promise for identifying therapeutic targets and biomarkers.

In this study, differential analysis of the GSE20592 cervical cancer dataset from the GEO database identified has-miR-125b as a key differentially expressed miRNA in cervical squamous cell carcinoma, positioned centrally within regulatory networks. Prediction tools revealed a binding site for miR-125b within the TAZ 3’UTR. MiR-125b, a highly conserved miRNA, exhibits tissue- and organ-specific expression patterns. Its diverse functions depend upon upstream regulatory factors and downstream targets. It can play an important role in cancer development through various oncogenic pathways, and has become a novel marker for tumor diagnosis and treatment (16).

Studies such as those conducted by Spirina et al. have implicated miR-125b in the regulation of thyroid cancer progression through modulation of autophagy-related proteins (17). Similarly, Pan et al. demonstrated that miR-125b-5p inhibits the malignant phenotype of endometrial cancer cells by targeting MTFP1, thereby functioning as a tumor suppressor gene (18). In cervical cancer, several studies have reported downregulation of miR-125b expression in cancerous tissues compared to normal tissues, although the precise underlying mechanisms remain unclear (19–21). Therefore, assessing the upstream regulation of miR-125b by HPV16 E6 and its downstream targets assumes critical importance in elucidating the mechanisms governing the onset and progression of cervical cancer.

In summary, the current body of evidence remains insufficient to comprehensively explain the interplay among HPV16 E6, miR-125b, and TAZ, as well as the precise contribution of miR-125b to cervical cancer. Therefore, the present study utilized a combination of bioinformatics analysis and dual-luciferase reporter gene assays to assess the potential role of miR-125b in cervical cancer and its targeting relationship with TAZ. Furthermore, cellular experiments were conducted to probe the regulatory interactions between HPV16 E6 and miR-125b, as well as the impact of miR-125b on the cellular physiological processes implicated in cervical cancer. The objective of this study is to provide insights on the potential mechanisms underlying the involvement of miR-125b in cervical cancer.

## 2 Materials and methods

### 2.1 Clinical data

From May 2022 to May 2023, cancer tissue samples were collected from 15 patients with HPV16 positive cervical squamous cell carcinoma (SCC, stages IA-IIA) at the gynecology outpatient clinic of the Second Hospital of Shanxi Medical University. This included 3 cases at stage IA2, 10 cases at stage IB, and 2 cases at stage IIA1. Pathological classification included 8 cases of highly differentiated carcinoma, 5 cases of moderately differentiated carcinoma, and 2 cases of poorly differentiated carcinoma. Additionally, lesion tissues from 15 patients with HPV16 positive high-grade squamous intraepithelial lesion (HSIL) were collected, comprising of 5 cases of CIN2 and 10 cases of CIN3, along with cervical tissues from 10 patients with chronic cervical inflammation. All tissue samples were assessed pathologically. The age group of the patients ranged from 30 to 60 years, with an average age of 45.8 ± 6.39 years. Following age-matching (± 3 years), samples from 5 patients with cervical squamous cell carcinoma, 5 patients with HSIL, and 5 patients with chronic cervical inflammation were selected, resulting in a total of 15 samples (refer to **Table 1**). Samples were immediately transferred to a -80°C ultra-low temperature freezer post-surgery. Exclusion criteria encompassed pregnant women, patients with other malignant tumors, patients who had undergone radiotherapy or chemotherapy prior to surgery, and those with a history of treatment for cervical or vaginal lesions. All participants in this study provided informed consent, and all clinical data and research findings were treated confidentially and used solely for this study, which was approved by the Ethics Committee of the Second Hospital of Shanxi Medical University.

**Table 1:** Clinicopathologic information of miRNA validation test specimens.

Group	Pathological typing	Age	TCT
1	SCC	45	HSIL
1	SCC	48	ASC-H
1	SCC	45	ASCUS
1	SCC	42	HSIL
1	SCC	46	SCC
2	CIN2	46	ASCUS
2	CIN3	46	ASCUS
2	CIN3	44	ASCUS
2	CIN3	45	ASC-H
2	CIN3	43	ASCUS
3	NC	45	NILM
3	NC	47	NILM
3	NC	44	NILM
3	NC	43	NILM
3	NC	46	NILM

SCC, cervical squamous cell carcinoma; CIN2, cervical intraepithelial neoplasia 2; CIN3, cervical intraepithelial neoplasia 3; NC, normal control; HSIL, High-grade squamous intraepithelial lesion; ASC-H, cannot exclude high-grade squamous intraepithelial lesion; ASCUS, atypical squamous cells of undetermined significance; NILM, negative for intraepithelial lesion or malignancy; TCT, thinprep cytologic test.

### 2.2 Bioinformatics analysis techniques

The “DESeq2” R package was used to conduct differential analysis on the GSE20592 cervical cancer dataset retrieved from the GEO database (comparing tumor versus normal samples), with thresholds set for differentially expressed genes as |log2 FC| > 0.5 and *P* < 0.05. Leveraging gene expression profiles and grouping information, the “e1071” and “randomForest” R packages facilitated the implementation of support vector machine (SVM-REF, Support Vector Machine Recursive Feature Elimination) and random forest (RF) machine learning techniques, respectively, to identify key miRNAs. Subsequently, the PicTar online prediction tool was used to predict the target genes of these key miRNAs, and the Cytoscape 3.9.1 software was used to construct network regulation maps presenting the relationships between miRNAs and their target genes. Functional enrichment analysis of the selected key miRNA target genes was conducted with the R package “clusterProfiler.”

### 2.3 Cell culture

Cervical cancer SiHa cells were obtained from the Chinese Academy of Sciences (Shanghai, China). They were cultured in DMEM (Procell, catalogue number: PM150210) complete medium supplemented with 10% fetal bovine serum (ExCell Bio, catalogue number: 111323) and 1% antibiotics (Procell, catalogue number: PB180120) under standard conditions of 37°C and 5% CO_2_ in a CO_2_ incubator.

### 2.4 Cell transfection and grouping

SiHa cells in the logarithmic growth phase and exhibiting robust growth were seeded at a density of 5×10^3^ cells per well in a 96-well cell culture plate and incubated overnight at 37°C, 5% CO_2_. Two hours preceding transfection, the culture medium was replaced with serum-free DMEM. Then miR-125b mimic, miR-125b-mimic-negative control, miR-125b inhibitor, and miR-125b-inhibitor-negative control were transiently transfected into cells at a concentration of 20 nM using Lipofectamine™ 2000(Invitrogen,catalogue number:11668-019),following the manufacturer’s instructions. The experimental groups were delineated as follows: SiHa; SiHa + miR-125b inhibitor negative control (NC); SiHa + miR-125b inhibitor; SiHa + miR-125b mimic negative control (NC); and SiHa + miR-125b mimic. The empty plasmid and HPV16E6 overexpression plasmid were transfected according to the manufacturer’s instructions. The experimental groupings were as follows: control; vector; OE. All specific primers were purchased from Qingke Biotech (Beijing, China). The primer sequences used are enumerated in **Table 2**.

**Table 2:** Sequences of all primers in this study.

Name	Primer	Sequence (5’-3’)	Size
U6	Forward	CGCTTCGGCAGCACATATAC	90bp
	Reverse	AAATATGGAACGCTTCACGA	
Hsa-miR-125b	loop primer	GTCGTATCCAGTGCAGGGTCCGAGGTATTCGCACTGGATACGACTCACAAG	87bp
	F primer	TGCGCTCCCTGAGACCCTAACT	
	R primer	CCAGTGCAGGGTCCGAGGTATT	
miR-125b mimic		UCCCUGAGACCCUAACUUGUCA	
miR-125b mimic NC		UUCUCCGACGUGUCACGUTT	
miR-125b inhibitor		UCACAAGUUAGGGUCUCAGGGA	
miR-125b inhibitor NC		CAGUACUUUUGUGUAGUACAA	
Homo GAPDH	Forward	TCAAGAAGGTGGTGAAGCAGG	115bp
	Reverse	TCAAAGGTGGAGGAGTGGGT	
Homo TAZ	Forward	CTCCCACTTCTTCAGCTTGG	152bp
	Reverse	TCTGGTAGACGCCATCTCCT	
Homo TEAD	Forward	GATGATGCTGGGGCTTTTTA	172bp
	Reverse	GGGAGCGGTTTATTCGGTAT	
Homo E6	Forward	GAATGTGTGTACTGCAAGCA	248bp
	Reverse	CACAGTGGCTTTTGACAGTT	

### 2.5 Dual-luciferase assay

The pYr-MirTarget-Report plasmids for miR-125b target TAZ 3’UTR were constructed as pYr-MirTarget- Homo- TAZ-3’UTR containing the wild-type 3’UTR of TAZ and as pYr-MirTarget- Homo- TAZ-3’UTR-mut containing the mutant 3’UTR of TAZ. The pYr-MirTarget- Homo- TAZ-3’UTR vector and pYr-MirTarget- Homo- TAZ-3’UTR-mut vector contain Firefly luciferase and Renilla luciferase. 293T cells were seeded into 12-well plates, and each was co-transfected with pYr-MirTarget-Homo-TAZ-3’UTR vector or pYr-MirTarget-Homo-TAZ-3’UTR-mut vector and 50 nM miR-125b mimic or miR-125b mimic NC using Lipofectamine™ 2000(Invitrogen,catalogue number:11668-019), according to the manufacturer’s protocol. After 48h transfection, cells were assayed for both firefly and Renilla luciferase using the dual luciferase glow assay(Beyotime, catalogue number:RG027). Renilla luciferase activity was normalized to Firefly luciferase activity (Renilla LUC/Firefly LUC). Each experiment was performed at least three times.

### 2.6 CCK-8 cell proliferation assay

Cells were seeded at a density of at 5×10^3^ cells per well in a 96-well plate. At 0 hours, 24 hours, and 48 hours post-seeding, 10 μl of CCK-8 (MCE, catalogue number: HY-K0301) solution was added to each well. Following a 2-hour incubation period at 37°C, the absorbance at 450 nm was measured using a spectrophotometer. All experiments were conducted three times.

### 2.7 Cell apoptosis assay

Cells were harvested via trypsin digestion and subsequently washed twice with PBS, followed by centrifugation at 1200 rpm for 5 minutes. The assay was conducted in accordance with the instructions provided by the manufacturer with the Annexin V-FITC/PI cell apoptosis assay kit (KeyGEN BioTECH, Nanjing, catalogue number: KGA108), and the samples were subsequently analyzed using a flow cytometer. All experiments were repeated three times.

### 2.8 Cell scratch assay

A line was marked on the bottom of a 6-well plate using a marker. Cells were seeded into the 6-well plate until reaching a confluency of over 90%. Subsequently, a pipette tip was used to establish a vertical scratch along the marked line. Following three washes with PBS and the addition of serum-free medium, photographs were captured at 0 hours and 48 hours post-scratching. All experiments were repeated three times.

### 2.9 Cell invasion assay

A 100 μl solution of Matrigel at a final concentration of 1 mg/mL was applied to the upper Transwell chamber and then incubated at 37°C for 4 to 5 hours. Following incubation, 800 μl of complete medium containing 10% FBS was introduced into the lower Transwell chamber. Subsequently, 200 μl (3×10^5^/mL) of cell suspension was seeded into the upper chamber. After 24 hours of incubation, the chambers were rinsed with PBS, and the cells were fixed with 70% ethanol for 1 hour. Following fixation, cells were stained with 0.5% crystal violet for 20 minutes at room temperature. Following another wash with PBS, non-migrating cells present on the upper chamber were gently wiped off using a clean cotton swab, and the remaining cells were photographed under a microscope. All experiments were repeated three times.

### 2.10 Real-time quantitative reverse transcription PCR

Total RNA was extracted from cultured cells using Trizol reagent (Ambion, catalogue number: 15596-026), following the instructions provided by the manufacturer, which included DNase I treatment. cDNA was synthesized using Oligo (dT) and specific stem-loop primers with the HiScript^®^ II Q Select RT SuperMix for RT-qPCR(VAZYME, catalogue number:R233) added to a SYBR Green Master Mix (VAZYME, catalogue number: Q111-02). The relative expression of miR-125b was calculated using the 2^-ΔΔCt^ method with U6 as endogenous control. All specific primers were purchased from Qingke Biotech (Beijing, China). The primer sequences used are enumerated in **Table 2**. All experiments were repeated three times.

### 2.11 Western blot

Whole cells were collected and lysed using RIPA buffer (Beyotime, Shanghai, China, catalogue number: P0013B), followed by 30-min incubation on ice and 10-min centrifugation (12000 r/min; 4°C). Protein expression was determined with the use of the BCA protein assay kit (Beyotime, Shanghai, China, catalogue number: P0010). Subsequently, 40 μg of lysates were loaded onto gels for electrophoresis and subsequently transferred to PVDF membranes using a wet transfer method. Following transfer, membranes were blocked at room temperature for 1 hour and then incubated overnight at 4°C with primary antibodies targeting GAPDH (1:1000), TAZ (1:1000), E6 (1:500), and TEAD (1:2000). Secondary antibodies were incubated on a shaker at room temperature for 2 hours. Protein detection was carried out using enhanced chemiluminescence, and grayscale values were analyzed using IPP. Replications of all experiments were conducted three times.

### 2.12 Protein-protein binding prediction

Protein pre-processing was conducted using Discovery Studio software, encompassing the elimination of water molecules, addition of hydrogens and charges, and extraction of the native ligand from the structure. Subsequently, the interaction between HPV16 E6 and TAZ was analyzed using PyMOL. Protein-protein docking was executed by using the ZDOCK module within Discovery Studio. The algorithm used fast Fourier transform-based correlation techniques to assess translational and rotational space within the protein-protein system. The docking sampling parameters were configured as follows: Angular Step Size set to 6 with a sampling angle of 15° to enhance prediction accuracy. The RMSD Cutoff was established at a cluster radius of 10.0 Å, while the Interface Cutoff was also set at 10.0 Å. Additionally, a maximum of 100 clusters were specified to optimize clustering outcomes. Subsequently, tools were used to enumerate the top 100 poses with higher ZDOCK scores, plotting ZDOCK score on the x-axis and clusters on the y-axis for enhanced visualization. Key poses exhibiting a ZDOCK score surpassing 13 were subsequently earmarked for further RDOCK docking analysis.

### 2.13 Statistical methods

Data analysis was conducted using R version 4.1.2 and SPSS version 22.0 statistical software packages. Quantitative data are presented as mean ± standard deviation (X ± S). The comparison of means between two groups was executed using the independent sample t-test, while comparisons among multiple groups were carried out with one-way analysis of variance (ANOVA), with a significance threshold set at *P* < 0.05.

## 3 Results

### 3.1 Screening differentially miRNA expression profiles in cervical cancer and bioinformatics analysis

#### 3.1.1 Twenty-eight differentially expressed miRNAs were found in cervical cancer

**Figure 1:**
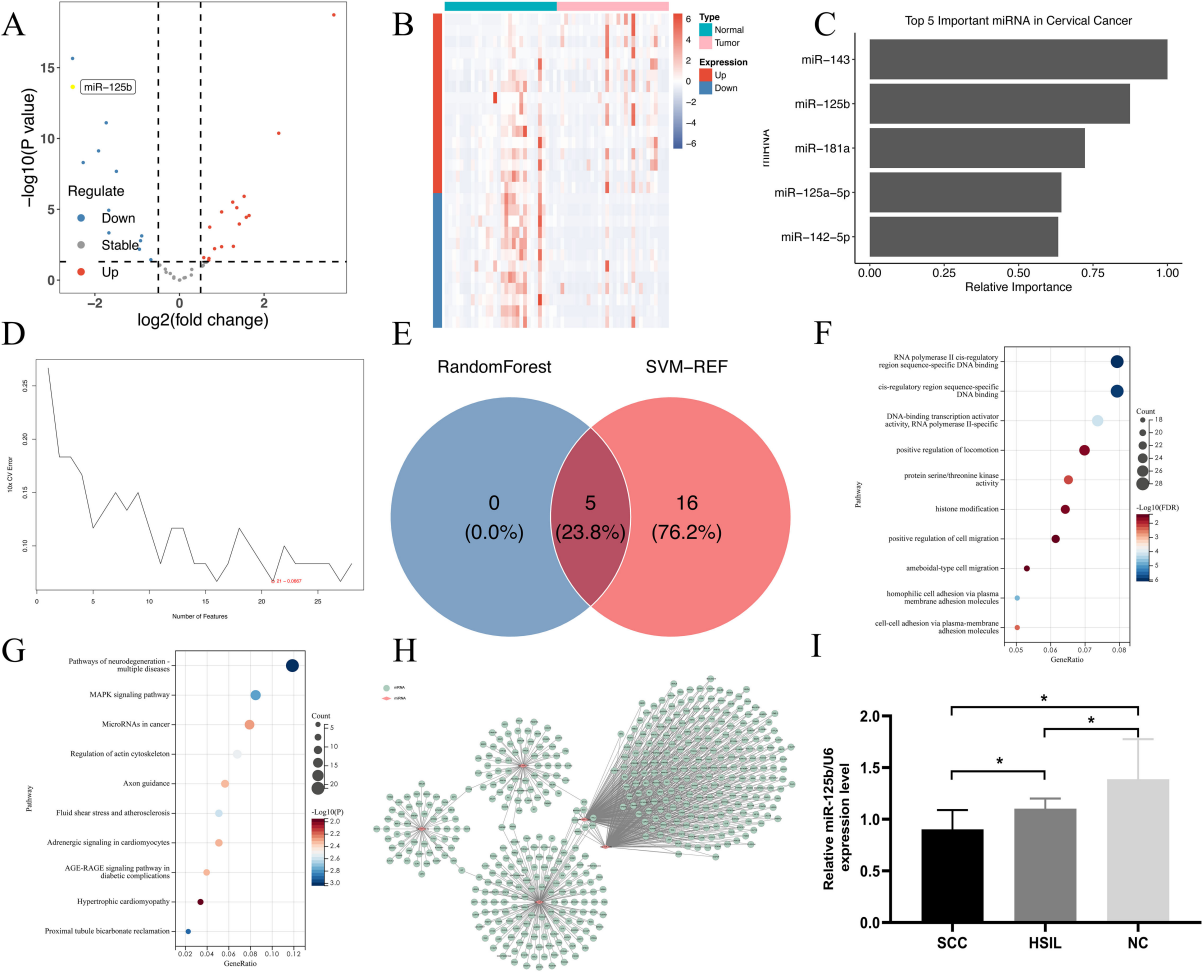
miR-125b is down-regulated in cervical cancer. **(A)** Volcano map for differentially expressed miRNAs in cervical cancer. Each dot in the figure represents a miRNA, and the blue and red dots represent significantly differentially expressed miRNAs. Moreover, the red dots indicate that the miRNA expression is up-regulated; the blue dots indicate that the miRNA expression is down-regulated, and the gray dots indicate that there is no significant difference in these miRNAs; **(B)** Heatmap for differentially expressed miRNAs in cervical cancer. Each small square represents a miRNA, and its color represents the expression level. The larger the expression level, the darker the color (red for high expression, blue for low expression). The first row represents sample groups, cyan represents Normal samples, and pink represents Tumor samples. Each row represents the expression level of each miRNA in different samples. The first column represents the grouping of differential miRNAs, red represents up-regulated miRNAs in Tumor samples compared to Normal samples, and blue represents down-regulated miRNAs; **(C)** Relative importance plot of the top 5 microRNAs in random forest; **(D)** Error rate of miRNA in 10-fold cross-validation of Support Vector Machine; **(E)** Intersection of key microRNAs screened by two machine learning methods; **(F)** GO enrichment pathway; **(G)** KEGG enrichment pathway; **(H)** miRNA-mRNA-Network. The miRNA gene interaction network using the attribute relationship between different miRNA and target gene. The pink nodes indicate miRNAs, the green nodes indicate targeting gene mRNA. The thick lines represent the interaction between miRNAs and gene. Centrality refers to the contribution of miRNA to surrounding gene or gene to surrounding miRNA; **(I)** The expression level of miR-125b from normal, HSIL, and SCC tissues (n=15) was verified by RT-qPCR. SCC: *P <0.05, compared with the normal group.

We screened differentially expressed miRNAs in cervical cancer using the GSE20592 database, and identified a total of 28 miRNAs. Among these, 16 exhibited upregulation, comprising of miR-142-5p, miR-21, miR-27a, miR-181a, miR-191, let-7i, miR-15b, miR-185, miR-423-5p, let-7b, miR-145, miR-335, let-7a, miR-19b, miR-23a, and let-7e; conversely, 12 were found to be downregulated, encompassing miR-143, miR-125b, miR-10b, miR-125a-5p, miR-424, miR-126, miR-100, miR-451, miR-99b, miR-23b, miR-99a, and miR-199a-5p (refer to **Figures 1A, B**).

#### 3.1.2 Screening of five key miRNAs

Initially, a machine learning analysis using random forest was conducted on the expression profiles of the various miRNAs, leading to the identification of top five characteristic miRNAs (refer to **Figure 1C**). Subsequent analysis using support vector machine recursive feature elimination (SVM-RFE) revealed the top 21 characteristic miRNAs (refer to **Figure 1D**). By intersecting these outcomes, the final set of key miRNAs were determined to be hsa-miR-143, hsa-miR-125b, hsa-miR-125a-5p, hsa-miR-181a, and hsa-miR-142-5p (refer to **Figure 1E**).

#### 3.1.3 GO functional and KEGG pathway enrichment analyses of key miRNAs’ network and signal transduction pathways

GO enrichment analysis of the key miRNAs revealed that their target genes were predominantly associated with biological processes such as RNA polymerase II cis-regulatory region sequence-specific DNA binding, protein serine/threonine kinase activity, DNA-binding transcription activator activity, histone modification, and positive regulation of motility (see **Figure 1F**). In KEGG enrichment analysis, the target genes exhibited enrichment across 249 pathways, with prominent involvement in the MAPK signaling pathway, miRNAs in cancer, and pathways associated with neurodegenerative diseases-multiple diseases (refer to **Figure 1G**).

#### 3.1.4 Key miRNA target gene prediction

Target gene prediction was conducted for the key miRNAs, and by using Cytoscape, a network regulation map of miRNAs and their target genes was established. It was observed that miR-125b occupies a central position within the network of differentially expressed miRNAs governing regulation (see **Figure 1H**).

#### 3.1.5 Expression of miR-125b in different cervical tissues

Given the potential association between miR-125b and the progression of cervical lesions, we assessed the expression levels of miR-125b across various cervical lesion tissues. The findings demonstrated a notable reduction in the expression levels of miR-125b in HSIL and SCC tissues when compared to normal cervical tissue (*P* < 0.05) (refer to **Figure 1I**), aligning with the conclusions drawn from the bioinformatics analysis.

In this study, 28 differentially expressed miRNAs in cervical cancer were screened from the GSE20592 database, and 5 key miRNAs were obtained through bioinformation analysis, namely, hsa-miR-143, hsa-miR-125b, hsa-miR-125a-5p, hsa-miR-181a, hsa-miR-142-5p. Meanwhile, through functional analysis of key miRNAs and target gene prediction, It was observed that miR-125b occupies a central position within the network of differentially expressed miRNAs governing regulation. Further detection of the expression level of miR-125b in different cervical tissues showed that compared with normal cervical tissues, the expression level of miR-125b in HSIL and SCC was significantly decreased.

### 3.2 Reduced expression of miR-125b enhances proliferation and migration of SiHa cells

**Figure 2:**
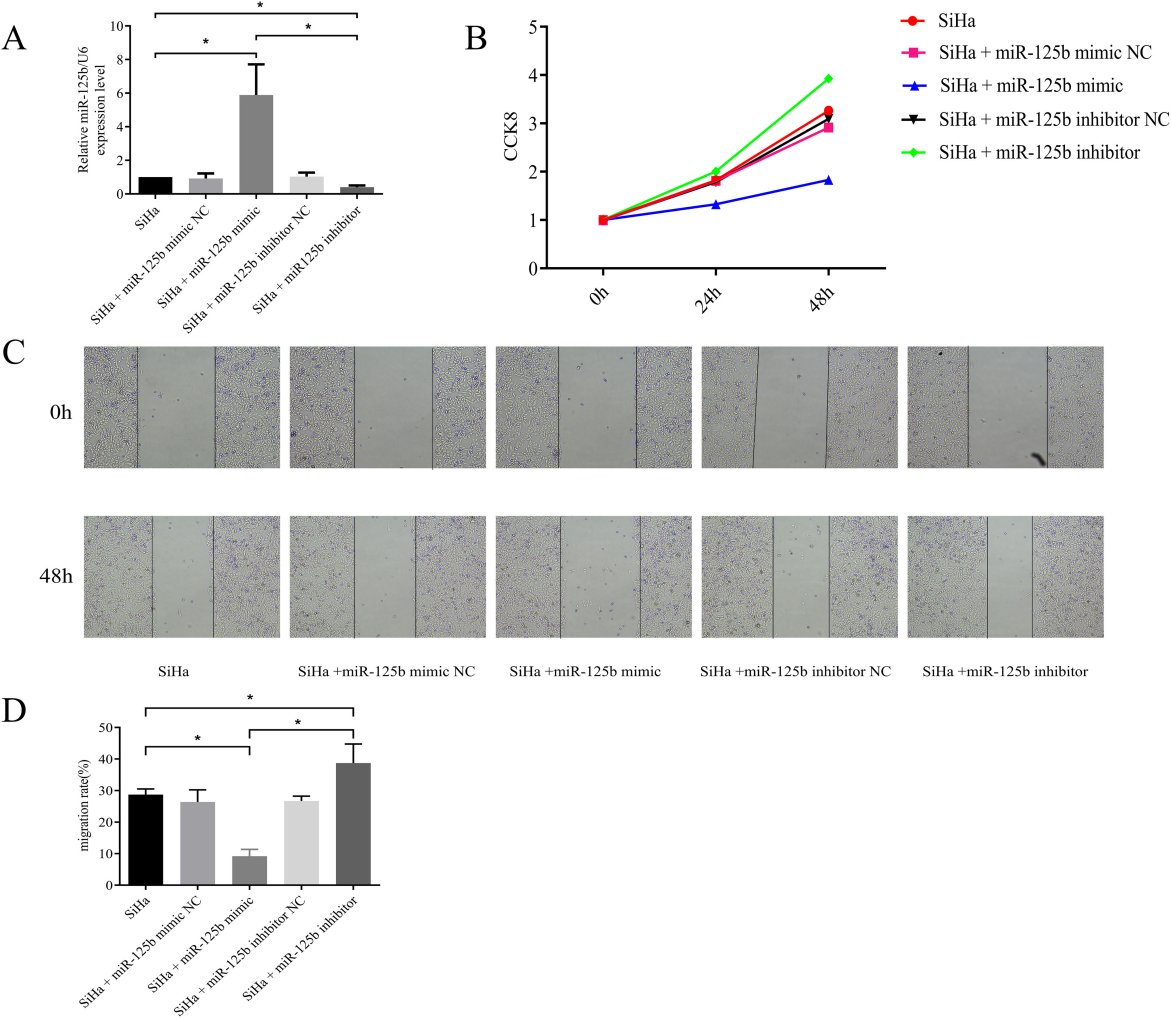
Low expression of miR-125b can promote proliferation and migration of SiHa cells. **(A)** SiHa cells were transfected with miR-125b mimic negative control, miR-125b mimic, miR-125b inhibitor negative control, and miR-125b inhibitor, and the expression level of miR-125b was estimated by RT-qPCR using U6 for normalization. miR-125b mimic increased miR-125b expression in SiHa cells, conversely miR-125b inhibitor decreased miR-125b expression; **(B)** Effect of miR-125b on SiHa cells proliferation by CCK8; **(C, D)** Wound healing assays were used to evaluate migration in the miR-125b-altered cells. n=3, *P< 0.05.

Cell models were developed using miR-125b mimic and inhibitor constructs. RT-qPCR results revealed significantly heightened expression of miR-125b in the miR-125b mimic group compared to the miR-125b mimic NC group (*P* < 0.0001), while notably reduced miR-125b expression was observed in the miR-125b inhibitor group (*P* < 0.0001) (refer to **Figure 2A**). Furthermore, cell proliferation was reduced in the miR-125b mimic group when compared to the NC group (*P* < 0.0001), whereas increased proliferation was evident in the miR-125b inhibitor group (*P* = 0.0007) (refer to **Figure 2B**). Scratch assay results demonstrated reduced cell migration in the miR-125b mimic group when compared to the NC group (*P* = 0.007), while increased migration was observed in the miR-125b inhibitor group when compared to the NC group (*P* = 0.0068) (refer to **Figures 2C, D**).

Overexpression of miR-125b can suppress proliferation and migration of SiHa cells.

### 3.3 miR-125b can suppress invasion and promote apoptosis of SiHa cells

**Figure 3:**
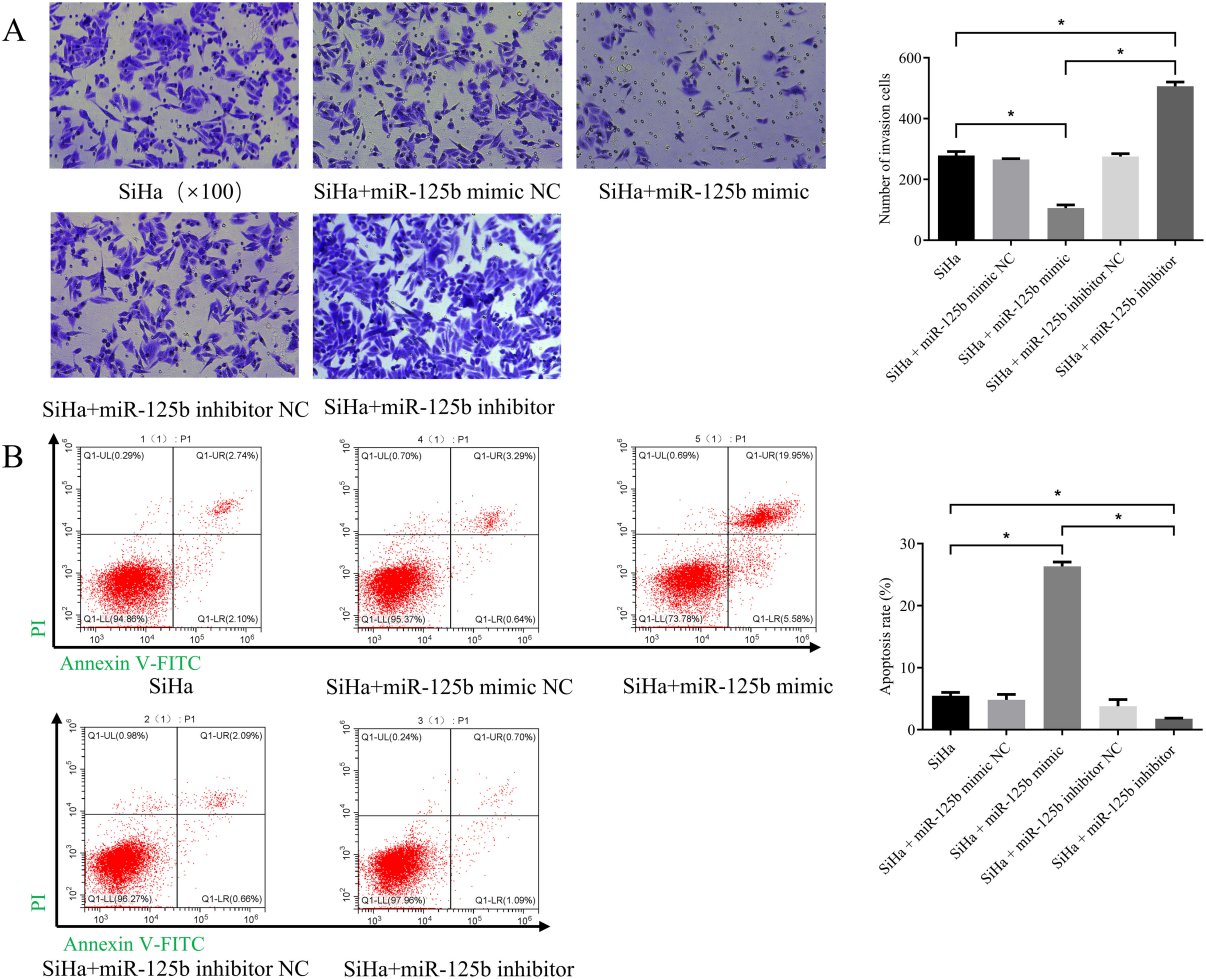
Low expression of miR-125b promotes the invasion of SiHa cells and suppresses cell apoptosis. **(A)** Transwell invasion assay were used to evaluate migration in the miR-125b-altered cells (scale bar=100μm); **(B)** Cell apoptosis assay was indicated that over-expression of miR-125b promotes apoptosis in SiHa cells. n=3, *P< 0.05.

We observed a reduction in cell invasion in the miR-125b mimic group when compared to the NC group (*P* < 0.0001), while an increase in invasion was noted in the miR-125b inhibitor group when compared to the NC group (*P* < 0.0001) (refer to **Figure 3A**). Additionally, cell apoptosis assay revealed a higher proportion of apoptosis in cells within the miR-125b mimic group compared to the miR-125b mimic NC group (*P* < 0.0001). Conversely, the miR-125b inhibitor group exhibited a lower apoptosis rate when compared to the miR-125b inhibitor NC group, with statistically significant differences (*P* = 0.0304; refer to **Figure 3B**).

Overexpression of miR-125b can suppress invasion and promote apoptosis of SiHa cells.

### 3.4 TAZ is a direct target of miR-125b

**Figure 4:**
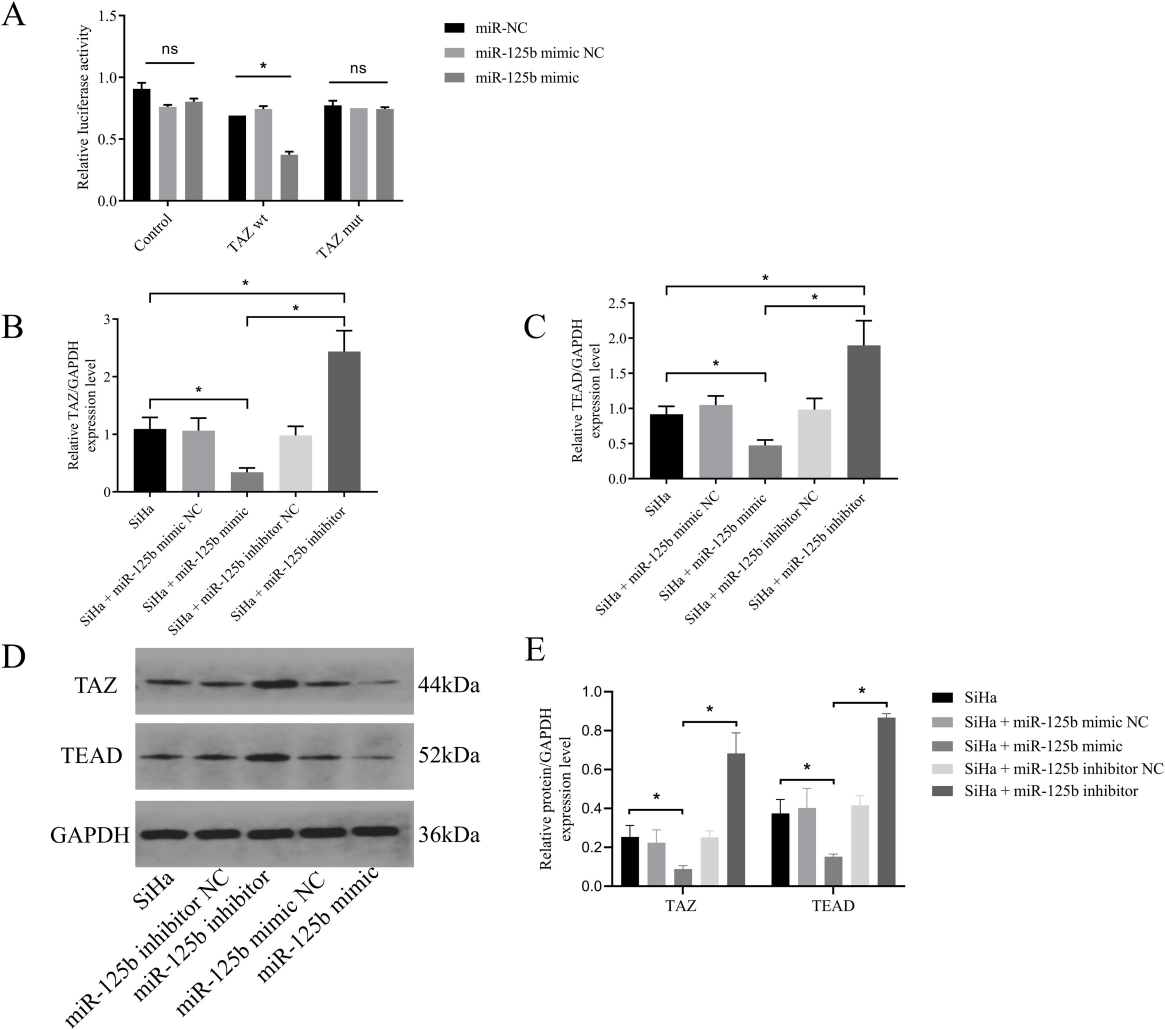
TAZ is a target gene of miR-125b. **(A)** Luciferase activity was measured in cells co-transfected with luciferase reporter containing the TAZ 3′-UTR and miR-125b mimic or mimic NC; **(B, C)**. SiHa cells were transfected with miR-125b mimic negative control, miR-125b mimic, miR-125b inhibitor negative control, and miR-125b inhibitor, and the expression level of TAZ mRNA and TEAD mRNA were estimated by RT-qPCR using GAPDH for normalization; **(D, E)** Western blot validation of TAZ and TEAD protein expression in SiHa cells after transfecting miR-125b mimic, miR-125b inhibitor, and their controls with GAPDH as the loading control. n=3, ns, not significant, *p < 0.05.TAZ wt: TAZ wild type.TAZ mut: TAZ mutant.

PicTar was used to predict the target genes of miR-125b, revealing 311 targets, with TAZ exhibiting a binding score of 137. To further validate the targeting interaction between miR-125b and TAZ, a dual-luciferase assay was conducted. Upon transfection of miR-125b mimic into SiHa cells, a significant reduction in luciferase activity was observed for TAZ wild type (wt) (*P* < 0.0001), while the luciferase activity of TAZ mutant (mut) exhibited no significant change (*P* = 0.4918) (refer to **Figure 4A**). Subsequently, RT-qPCR results revealed a reduction in TAZ mRNA expression post-transfection with miR-125b mimic when compared to the miR-125b mimic NC group (*P* < 0.0001). Conversely, the miR-125b inhibitor group displayed an increase in TAZ mRNA expression when compared to the miR-125b inhibitor NC group (*P* < 0.0001) (refer to **Figure 4B**). Subsequent, TEAD that is the downstream gene of TAZ was detected. RT-qPCR results demonstrated decreased expression of TEAD mRNA (*P* < 0.0001) in the miR-125b mimic group when compared to the miR-125b mimic negative control (NC) group, whereas the miR-125b inhibitor group exhibited increased expression of TEAD mRNA when compared to the miR-125b inhibitor NC group (*P* < 0.0001) (refer to **Figure 4C**). Western blot analysis further validated these findings, indicating increased protein expression of TAZ (*P* = 0.0025) and TEAD (*P* = 0.0002) in the miR-125b inhibitor group compared to the miR-125b inhibitor NC group. Conversely, reduced expression of TAZ (*P* = 0.0286) and TEAD (*P* = 0.0130) was observed in the miR-125b mimic group when compared to the miR-125b mimic NC group (refer to **Figures 4D, E**).

These results confirmed the direct binding between miR-125b and TAZ mRNA. In addition, the expression of miR-125b was negatively correlated with the expression of TAZ and TEAD.

### 3.5 Role of HPV16 E6 in miR-125b targeting TAZ to regulate the Hippo pathway

**Figure 5:**
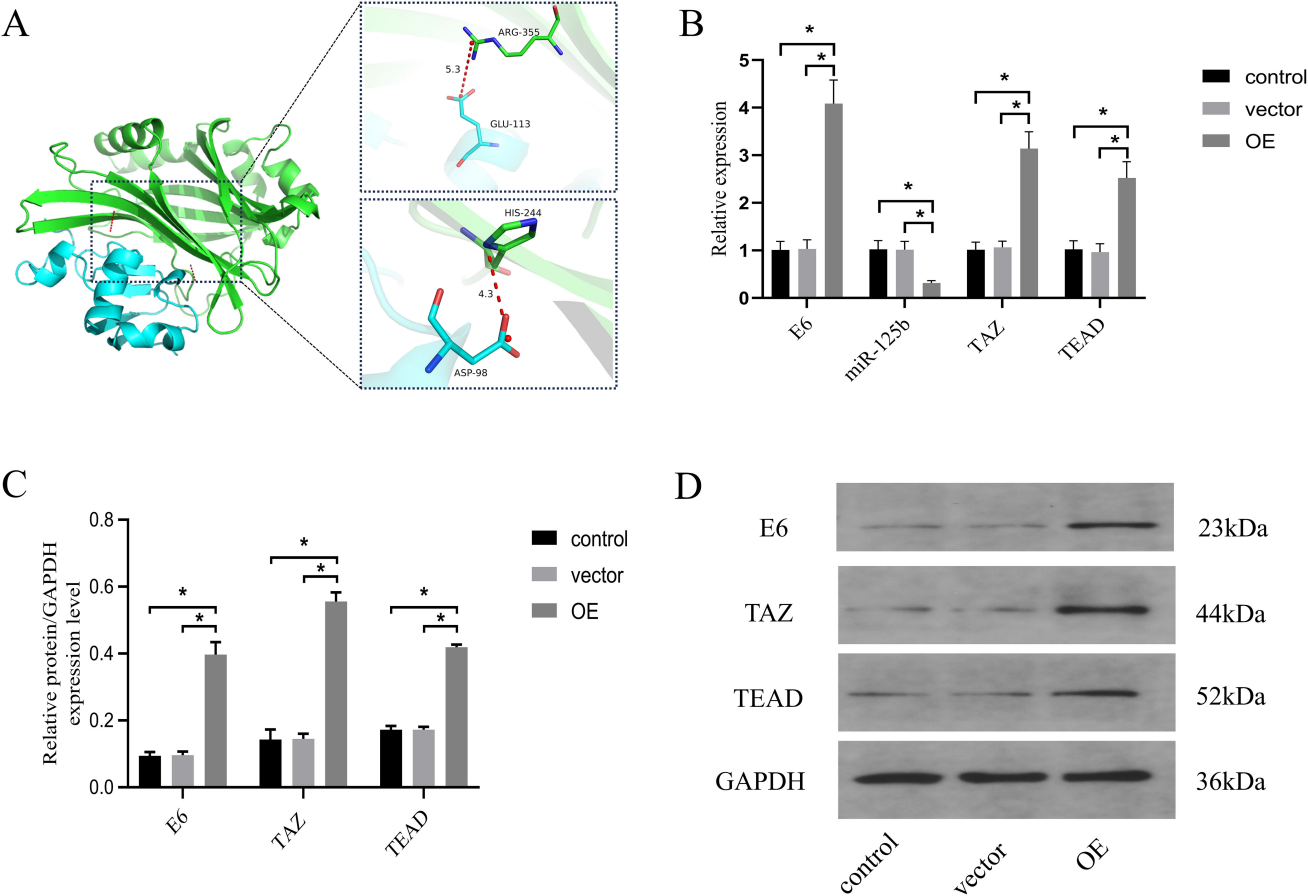
miR-125b/TAZ regulates the Hippo pathway through interaction with HPV16 E6. **(A)** protein-protein interaction prediction revealed that ARG355 and HIS244 of TAZ formed salt bridge interactions with ASP98 and GLU113 of HPV16 E6 protein. Blue color represents HPV16 E6 protein, green color represents TAZ protein; **(B)** SiHa cells were overexpressed E6 and the expression level of miR-125b, TAZ mRNA and TEAD mRNA were estimated by RT-qPCR; **(C, D)** Western blot validation of TAZ and TEAD protein expression in SiHa cells after overexpression E6 with GAPDH as the loading control. n=3, *p < 0.05. OE, over expression of HPV16 E6.Vector: transfection of empty plasmids.

To assess the regulatory interplay among HPV16 E6, miR-125b, and the Hippo pathway, we preliminarily explored the relationship between HPVE6 and TAZ firstly. Protein-protein interaction prediction revealed that the ARG355 and HIS244 of TAZ formed salt bridge interactions with the ASP98 and GLU113 of the HPV16 E6 protein, with hydrogen bond distances of 4.3 and 5.3 angstroms, respectively (refer to **Figure 5A**).

Next, SiHa cells were transfected with an HPV16 E6 overexpression plasmid and an empty vector. Comparative analysis revealed significantly increased expression levels of HPV E6 mRNA, TAZ mRNA, and TEAD mRNA (*P* < 0.05), accompanied by a notable reduction in miR-125b expression levels (*P* < 0.05) in the overexpression (OE) group when compared to the control and vector groups (refer to **Figure 5B**). Western blot analysis further corroborated these findings, demonstrating a pronounced increase in the protein expression of HPV 16 E6 (*P* = 0.0002), TAZ (*P* < 0.0001), and TEAD (*P* < 0.0001) in the OE group when compared to the control and vector groups (refer to **Figures 5C, D**).

These results suggest that HPV E6 may promote the progression of cervical cancer by inhibiting the expression level of miR-125b targeting TAZ/TEAD.

## 4 Discussion

Cervical cancer ranks as the fourth most prevalent cancer affecting women globally. While advancements in screening methods and vaccination initiatives in developed countries have been notable, a concerning trend has emerged with a widening gap in disease burden between women in developed nations and those in resource-limited countries. Currently, a staggering 85% of cervical cancer deaths occur in low- and middle-income countries (22). This disparity is particularly pronounced in China, where cervical cancer has become a leading cause of cancer-related death among women. Hence, assessing the pathogenesis of cervical cancer and devising strategies to block its progression are vital endeavors demanding immediate attention.

Persistent infection with high-risk HPV is a prerequisite for the development of cervical cancer. Notably, patients with HPV16-positive low-grade squamous intraepithelial lesion (LSIL) face a 10% risk of transitioning to HSIL within a four-year span, highlighting HPV16 as the foremost genotype driving cervical lesion progression. This process predominantly revolves around the upregulation of the early protein E6 within host cells of HPV, which instigates various defensive responses, such as the suppression of tumor suppressor genes and activation of oncogenes implicated in cancer metastasis pathways, thus fostering the malignant transformation of host cells (23). Recent studies have revealed the ability of the E6 oncoprotein of HPV to upregulate DNA methyltransferases, precipitating aberrant methylation of miRNAs and subsequently influencing microRNA expression patterns in cervical cancer (24). Therefore, assessing dysregulated miRNAs in cervical cancer holds substantial promise for discerning potential therapeutic targets and biomarkers. In this context, we leveraged bioinformatics analyses to scrutinize differentially expressed miRNAs in cervical squamous cell carcinoma sourced from the GEO database, pinpointing five characteristic miRNAs: hsa-miR-143, hsa-miR-125b, hsa-miR-125a-5p, hsa-miR-181a, and hsa-miR-142-5p. Further functional analysis revealed that miR-125b assumes a pivotal role within the network of differentially expressed miRNAs governing regulatory processes, exerting substantial influence over the progression of cervical cancer.

MiR-125b, a miRNA recently identified and highly conserved across mammals, vertebrates, and nematodes, plays a crucial role in various cellular processes including cell proliferation, apoptosis, differentiation, and embryonic development through its targeting of multiple proteins (25). Recent studies have revealed dysregulated expression of miR-125b in diverse malignancies. For instance, it exhibits upregulation in colorectal and gastric cancers, where it emerges as a potential diagnostic and therapeutic biomarker (26, 27). Conversely, in ovarian cancer, miR-125b experiences downregulation and exerts negative modulation on CD147 expression, thereby facilitating tumorigenesis and inhibiting apoptosis (28). This signifies the contextual duality of the functionality of miR-125b, either as oncogenic or anti-cancerous, contingent upon the specific cancer subtype, but with limited assessment in cervical cancer.

To assess the potential involvement of miR-125b in cervical cancer progression, we validate its expression patterns in different cervical tissues based on bioinformatics analyses. Results revealed a significant reduction in miR-125b expression levels in cervical squamous cell carcinoma and HSIL specimens compared to normal cervical tissue, demonstrating statistically significant differences (*P* < 0.05). Also, following successful transfection of the HPV16-positive cervical cancer cell line SiHa with either miR-125b mimic or inhibitor, it was discerned that differential miR-125b expression profoundly influenced various biological behavior of SiHa cells pertinent to proliferation and migration. Specifically, transfection of miR-125b mimic increased the miR-125b expression levels in SiHa cells, precipitating a significant reduction in cell proliferation, migration and invasion capacities, but increased apoptotic capacity Conversely, transfection of miR-125b inhibitor in SiHa cells downregulated miR-125b expression, consequently instigating a notable enhancement in cell proliferation, migration and invasion capabilities, but decreased apoptotic capacity. These observations underscore the tumor-suppressive role of miR-125b in cervical cancer development. While these findings align with studies conducted by Wang (29) and Hu et al. (21), they diverge from those reported by Sun et al. (30) Therefore, further research is warranted to delineate the precise mechanistic pathways through which miR-125b operates in cervical cancer, as well as to clarify its diagnostic and therapeutic implications in the disease context. Additionally, the potential regulatory influence of HPV E6 on miR-125b expression remains to be determined.

To explore the specific role of miR-125b in cervical cancer, bioinformatics analysis was utilized for predictive modeling and subsequently TAZ was validated as a target of miR-125b through dual-luciferase reporter gene assays. Also, the results of both RT-qPCR and western blot experiments corroborated that overexpression of miR-125b suppresses the expression of TAZ mRNA and protein, whereas suppression of miR-125b fosters the upregulation of TAZ mRNA and protein levels. TAZ, a transcriptional co-activator situated at the distal locus of chromosome Xq28 and comprising approximately of 400 amino acids, encompasses a WW domain and a PDZ binding domain. Operating as a pivotal effector within the Hippo signaling cascade, TAZ manifests dysregulated expression across various cancers, wherein it undergoes nuclear translocation and functions as an oncogene. Notably, in cervical cancer, studies have revealed a correlated increase in TAZ protein expression commensurate with the severity of cervical lesions. TAZ can target PD-L1, induce cervical cancer cell proliferation and metastasis, and inhibit apoptosis. However, the precise upstream regulatory mechanisms governing TAZ remain unclear (31).

miRNAs, serving as endogenous, non-coding, small single-stranded RNA entities, exert regulatory control over post-transcriptional gene expression, thereby bearing significant relevance to tumor development and progression. Numerous studies have underscored the direct targeting capabilities of miRNAs toward pivotal constituents of the Hippo pathway, thereby regulating tumor-associated signaling pathways and emerging as a focal point within tumor research.

However, previous studies have not documented miRNAs targeting TAZ in cervical cancer. This study is a pioneering effort to reveal the regulatory relationship between miR-125b and TAZ, elucidating the capacity of miR-125b to modulate TAZ expression levels, thereby impeding cervical cancer cell proliferation and migration while promoting apoptosis. Additionally, our findings reveal a correlated reduction in TEAD expression levels upon transfection with miR-125b mimic, and increased TEAD expression levels following transfection with miR-125b inhibitor. This indicates that miR-125b may regulate TAZ via the Hippo pathway, thereby hindering the progression of cervical cancer. However, whether its upstream is regulated by E6 remains to be confirmed.

To assess the potential involvement of HPV E6 in this process, we used protein-protein binding prediction to predict the relationship between HPV16 E6 and TAZ. The result indicated the potential for ARG355 and HIS244 from TAZ residues to form salt bridge interactions with specific residues of the HPV16 E6 protein, namely ASP98 and GLU113. This prediction is consistent with what Molly R Patterson et al. have said, TAZ is dysregulated in a HPV-type dependent manner (32). Furthermore, the expression levels of HPV E6, miR-125b, TAZ, and TEAD in cells transfected with an HPV E6 overexpression plasmid were compared to cells transfected with a control empty vector. The group with HPV E6 overexpression exhibited significantly increased levels of HPV E6, TAZ, and TEAD, along with a notable decrease in miR-125b expression. These findings provide compelling evidence indicating that HPV E6 might regulate miR-125b, thereby targeting TAZ via the Hippo pathway to hinder the progression of cervical cancer. Notably, this represents the first report of such a mechanism in cervical cancer. Previously, the role of HPV E6 was confined to downregulating miR-125b via the Wnt/β-catenin signaling pathway, thereby fostering cancer cell proliferation in esophageal cancer (15).

In conclusion, our study provides mechanistic insights into the role of HPV16 E6 in cervical cancer pathogenesis. We observed that HPV16 E6 exerts suppressive effects on miR-125b expression, leading to increased expression of its downstream target gene, TAZ, thereby influencing the Hippo pathway. These alterations promote cervical cancer cell proliferation, migration, and invasion while concurrently inhibiting apoptosis. These findings underscore the potential therapeutic significance of targeting miR-125b in the management of HPV-induced cervical cancer.

## Data Availability

The original contributions presented in the study are included in the article/supplementary material. Further inquiries can be directed to the corresponding author.
